# Data replicating the factor structure and reliability of commonly used measures of resilience: The Connor–Davidson Resilience Scale, Resilience Scale, and Scale of Protective Factors

**DOI:** 10.1016/j.dib.2016.08.001

**Published:** 2016-08-06

**Authors:** A.N. Madewell, E. Ponce-Garcia, S.E. Martin

**Affiliations:** a Southeastern Oklahoma State University, 215 Russell Hall, Durant, OK 74701, USA; b Cameron University, Lawton, OK, USA

**Keywords:** Scale of Protective Factors, Resilience Scale, Connor–Davidson Resilience Scale, Emerging adulthood, Confirmatory factor analysis

## Abstract

The data presented in this article are related to the article entitled “Assessing Resilience in Emerging Adulthood: The Resilience Scale (RS), Connor Davidson Resilience Scale (CD-RISC), and Scale of Protective Factors (SPF)” (Madewell and Ponce-Garcia, 2016) [1]. The data were collected from a sample of 451 college students from three universities located in the Southwestern region of the United States: 374 from a large public university and 67 from two smaller regional universities. The data from the three universities did not significantly differ in terms of demographics. The data represent participant responses on six measurements to include the Resilience Scale-25 (RS-25), Resilience Scale-14 (RS-14), Connor Davidson Resilience Scale-25 (CD-RISC-25), Connor Davidson Resilience Scale-10 (CD-RISC-10), Scale of Protective Factors-24 (SPF-24), and the Life Stressor Checklist Revised (LSC-R).

**Specifications Table**

**Table:** 

Subject area	*Psychology*
More specific subject area	*Lifespan Developmental Psychology*
Type of data	*Excel document, Figures*
How data was acquired	*Survey*
Data format	*Raw*
Experimental factors	*We assessed each participant for the occurrence of stressful and traumatic life events through the Life Stressor Checklist-Revised. This information was used in relation to resilience as assessed by five competing theoretical models.*
Experimental features	*We collected our data over a period of three semesters at three different universities in the Southwestern United States. The participants completed the survey either online or in a paper format within a research lab on campus.*
Data source location	*Southeastern Oklahoma State University, Durant, Oklahoma; Cameron University, Lawton, OK; Oklahoma State University, Stillwater, Oklahoma, United States of America.*
Data accessibility	*Data are available with this article.*

**Value of the data**

• This data provides a large sample of emerging adults from the Southwestern region of the USA, to include scores on three measures of resilience used in emerging adulthood, i.e., the Resilience Scale, the Connor–Davidson Resilience Scale, and the Scale of Protective Factors.
• The data could be used to assess and compare prevalence rates for resilience and specific stressful and traumatic events in emerging adult populations from different regions of the United States.
• The data includes information indicating the impact on daily living that stressful or traumatic events have had. This information could help guide researchers in comparing and assessing normative adjustment to such events.

## 1 Data

This article contains raw and coded data related to research published by Madewell and Ponce-Garcia [1]. We obtained the data through an online survey tool known as surveygizmo. We focused on students who were emerging adults, between the ages of 18–25 years of age, and enrolled at three different institutions across the state of Oklahoma, USA. The data is presented in an Excel-file (xls), which contains two data sheets: i) partially coded survey data and ii) codebook; containing the names for each item, the original references for each measure, and an explanation of scores.

## 2 Experimental design, materials and methods

The data were collected from a sample of 451 emerging adult college students from three universities located in the Southwestern region of the United States of America. Of the 451 emerging adults, 341 (75.6%) were women and 110 (24.4%) were men. The mean age was 19.1 (*SD*=1.39) and ages ranged from 18 to 25. The sample consisted of 74.3% White, 6% Asian–Asian Pacific Islander, 5.5% Black, 3.3% Native American, 2.4% Latino-Hispanic, 8.4% other or did not report ethnicity. The data from the three universities did not significantly differ in terms of demographic characteristics as the Pearson Chi-square was *X*^2^=.68, *df*=2, *p*=.71, *Cramer׳s V*=.039. Refer to Table 1 for more details about each sample.

### 2.1 Research design and measurement models

Psychological theory suggests that resilience develops in people who have protective factors that are more robust than their risk factors [2], [3]. Because of this theory, the dataset includes commonly used measures of resilience, the Resilience Scale-25 (RS-25) [4], Resilience Scale-14 (RS-14) [5], Connor Davidson Resilience Scale-25 (CD-RISC-25) [2], Connor Davidson Resilience Scale-10 (CD-RISC-10) [6], an assessment of protective factors known as the Scale of Protective Factors-24 (SPF-24) [7], and an assessment of stressful and traumatic events known as the Life Stressor Checklist Revised (LSC-R) [8].

### 2.2 Explanation of coding and figures

Participant responses for the RS-25 are coded using a 7-point Likert scale (1=*disagree,* 3=*neither agree nor disagree,* 7=*agree*), see Supplementary Fig. 1. Participant responses for the RS-14 are coded using a 7-point Likert scale (1=*disagree,* 3=*neither agree nor disagree,* 7=*agree*), see Supplementary Fig. 2. Participant responses for the CD-RISC-25 are coded using a 5-point Likert scale (0=*not true at all,* 4=*true nearly all of the time*), see Supplementary Fig. 3. Participant responses for the CD-RISC-10 are coded using a 5-point Likert scale (0=*not true at all,* 4=*true nearly all of the time)*, see Supplementary Fig. 4. Participant responses for the SPF-24 were coded using a 7-point Likert scale (1=*disagree completely,* 3=*neither agree nor disagree,* 7=*agree completely*), see Supplementary Fig. 5. The SPF-24 includes a hierarchic theoretical factor structure, containing two social/interpersonal protective factors that include social skills and social support, and two cognitive/individual protective factors that include goal efficacy and planning and prioritizing behavior. Refer to the model presented in Supplementary Fig. 5.

Data for the LSC-R represents the prevalence of stressful and traumatic events. For each of the first 29 items, the data was coded, either 1 for yes or 0 for no regarding whether the participant indicated having experienced the event in his or her lifetime. For endorsed items, the degree to which the participant felt the event had affected his or her daily life in the past year used a 5-point Likert scale (1=*not at all,* 3=*some,* 5=extremely). The 30th item on the LSC-R was an open-ended item that asked participants to report any significant stress or trauma he or she may have experienced in his or her life that was not already assessed in the first 29 items. Additionally, the data includes participant responses to demographic questions including age (ranging from 18 to 25 years), sex (1=*men,* 2=*women*), and ethnicity (1=*Black,* 2=*White, Non-Hispanic,* 3=*Hispanic/Latino,* 4=*Asian/Pacific Islander,* 5=*Native American,* 6=*mixed ethnicity*).

Supplementary Figs. 1–5 represent theoretical and statistical measurement models that were developed through a Structural Equation Modeling software known as Analysis Moment of Structures (AMOS 23.0). The models presented in Supplementary figures do not include the entire dataset, instead they focus on participants who indicated experience of significant stress or trauma (*n*=412). Supplementary Figs. 1 and 2 represent the model fit (as evaluated by the variance-covariance matrix) of the Resilience Scale items, with the RS-14 displaying somewhat improved covariates when compared to the RS-25. Similarly, Supplementary Figs. 3 and 4 represent the model fit of the Connor–Davidson Resilience Scale. When comparing Figs. 3 and 4, Fig. 4 demonstrates improved factor covariates. Supplementary Fig. 5 represents the model fit for the Scale of Protective Factors. The covariates demonstrate support for the use of the hierarchic design of the measure. Refer to Madewell and Ponce-Garcia [1] for more information on the analysis of theoretical and statistical model fit among these different measures of resilience. Additional replications with other samples would result in more parsimony within the field of resilience research.

## Figures and Tables

**Table 1:** Sample statistics.

**Responses**	Large public university	Southeastern regional university	Southwestern regional university	Total sample
**Sample size (*****N*****)**	**384**	**43**	**24**	**451**
Men (%)	91 (23.7%)	12 (27.9%)	7 (29.2%)	110 (24.4%)
Women (%)	293 (76.3%)	31 (72.1%)	17 (70.8%)	341 (75.6%)
Mean age (SD)	19.60 (1.38)	19.12 (1.37)	19.12 (1.36)	19.1 (1.39)

The researchers focused on an Emerging Adult sample, with ages ranging between 18 and 25 years.
