# Supplementary material for: Data replicating the factor structure and reliability of commonly used measures of resilience: The Connor–Davidson Resilience Scale, Resilience Scale, and Scale of Protective Factors
Source: Data Brief. 2016 Aug 6;8:1387–90. doi: 10.1016/j.dib.2016.08.001 (PMC4993855; doi:10.1016/j.dib.2016.08.001)
Supplement: Supplementary file 1 — Supplementary material [file mmc1.docx]

Conflicts of Interest Statement

Manuscript title: ***Data replicating the factor structure and reliability of commonly used measures of resilience: The Connor-Davidson Resilience Scale, Resilience Scale, and Scale of Protective Factors***

The authors whose names are listed immediately below certify that they have NO affiliations with or involvement in any organization or entity with any financial interest (such as honoraria; educational grants; participation in speaker’ bureaus; membership, employment, consultancies, stock ownership, or other equity interest; and expert testimony or patent-licensing arrangements), or non-financial interest (such as personal or professional relationships, affiliations, knowledge or beliefs) in the subject matter or materials discussed in this manuscript.

**Author names:**

1. Amy N. Madewell
2. Elisabeth Ponce-Garcia
3. Sarah E. Martin

The authors whose names are listed immediately below report the following details of affiliation or involvement in an organization or entity with a financial or non-financial interest in the subject matter or materials discussed in this manuscript. Please specify the nature of the conflict on a separate sheet of paper if the space below is inadequate. ..

**Author names:**

none

This statement is signed by all the authors to indicate agreement that the above information is true and correct:

**Author’s name type Author’s signature Date**

Amy N. Madewell
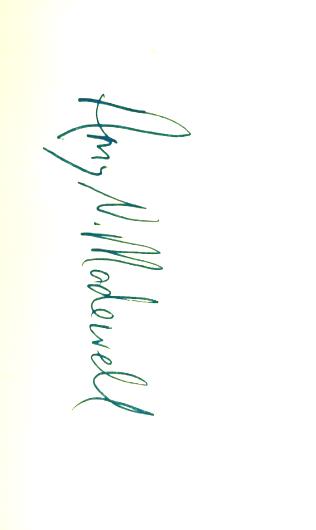
 5/18/2016

Elisabeth Ponce-Garcia
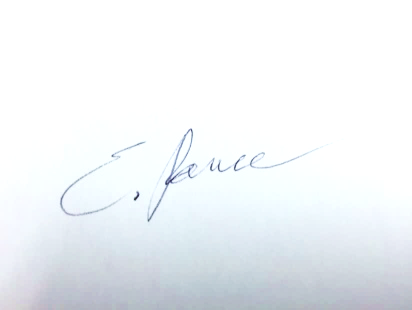
 5/18/2016

Sarah E. Martin 5/18/2016
