# Supplementary material for: Data replicating the factor structure and reliability of commonly used measures of resilience: The Connor–Davidson Resilience Scale, Resilience Scale, and Scale of Protective Factors
Source: Data Brief. 2016 Aug 6;8:1387–90. doi: 10.1016/j.dib.2016.08.001 (PMC4993855; doi:10.1016/j.dib.2016.08.001)
Supplement: Supplementary file 3 — Supplementary material [file mmc3.docx]

| Supplementary Figure 1. Factor Structure of Resilience Scale 25 | | | |
| --- | --- | --- | --- |
| **Error terms** | **Items** | **Covariates** | **Scale Name** |
| 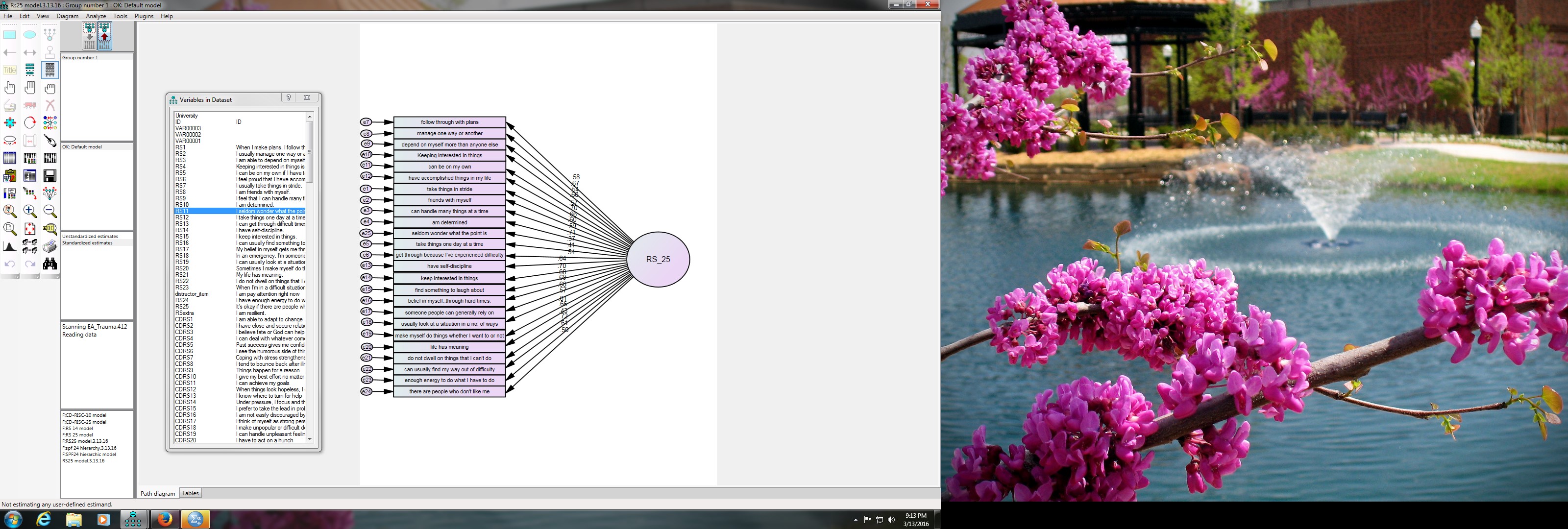 | | | |
| *Note.* Abbreviated items are presented in this table, for complete items refer to www.resiliencescale.com. | | | |

| Supplementary Figure 2. Improved Factor Structure of Resilience Scale 14 | | | |
| --- | --- | --- | --- |
| **Error terms** | **Items** | **Covariates** | **Scale Name** |
| 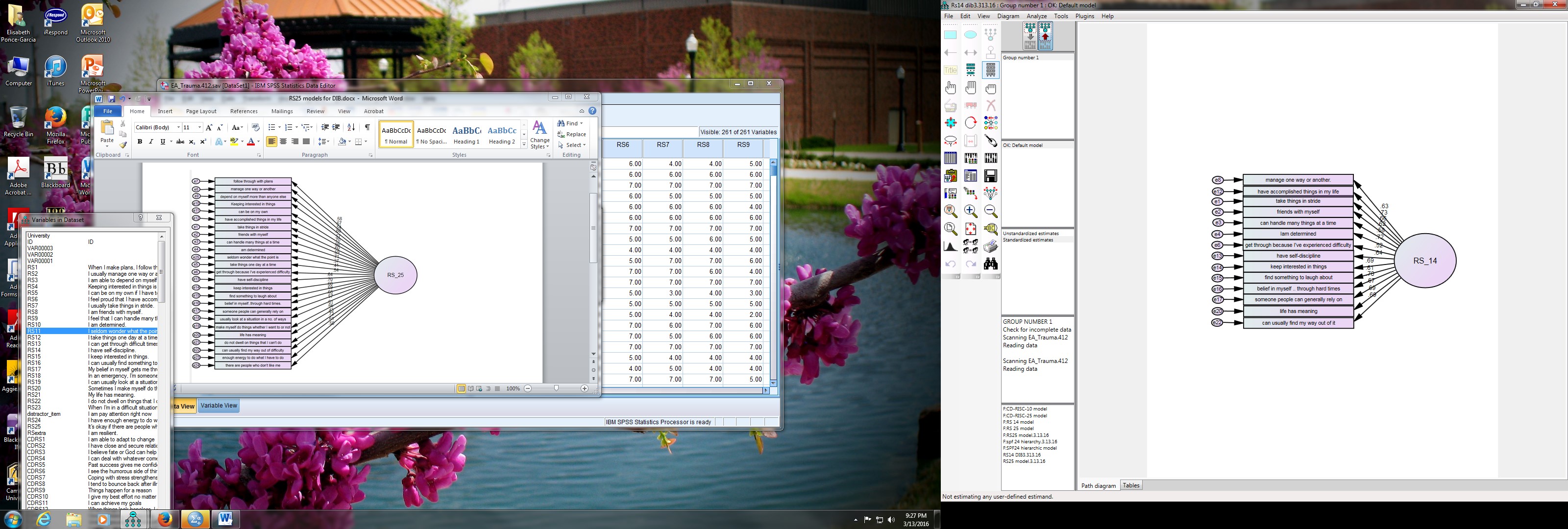 | | | |
| *Note.* Abbreviated items are presented in this table, for complete items refer to www.resiliencescale.com. | | | |

| Supplementary Figure 3. Factor Structure of the Connor-Davidson Resilience Scale 25 | | | |
| --- | --- | --- | --- |
| **Error terms** | **Items** | **Covariates** | **Scale Name** |
| 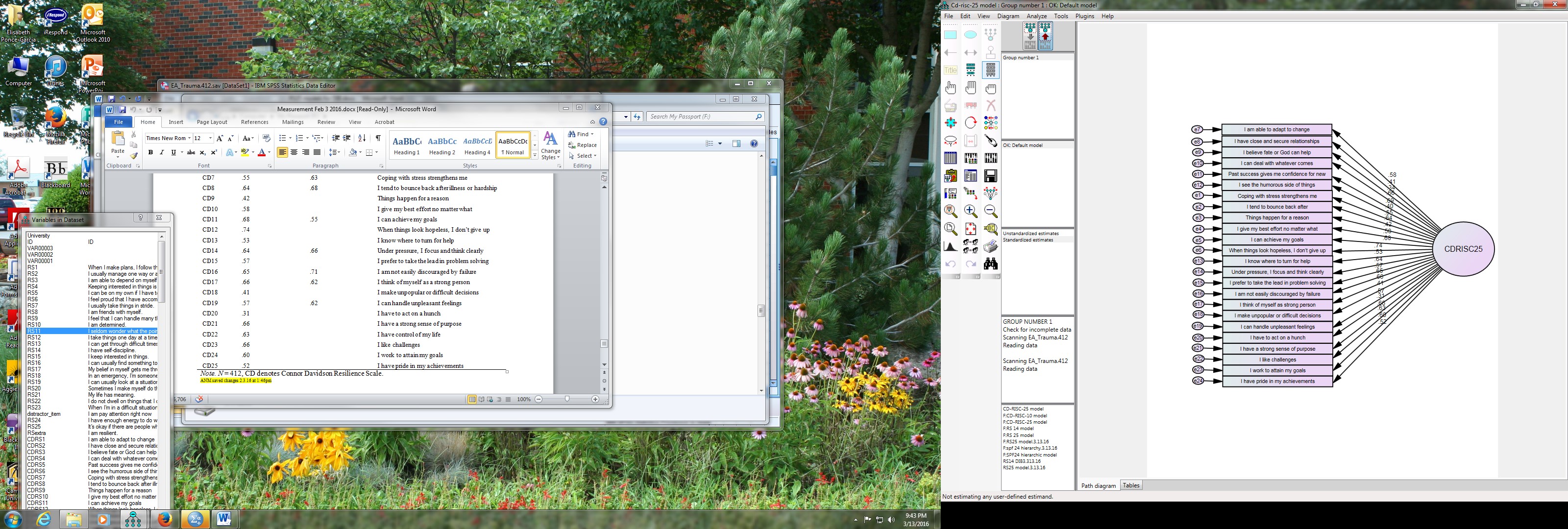 | | | |
| *Note.* Abbreviated items are presented in this table. To gain access to complete CD-RISC items, contact Dr. Jonathan Davidson at jonathan.davidson@duke.edu. | | | |

| Supplementary Figure 4. Factor Structure of the Connor-Davidson Resilience Scale 10 | | | |
| --- | --- | --- | --- |
| **Error terms** | **Items** | **Covariates** | **Scale Name** |
| 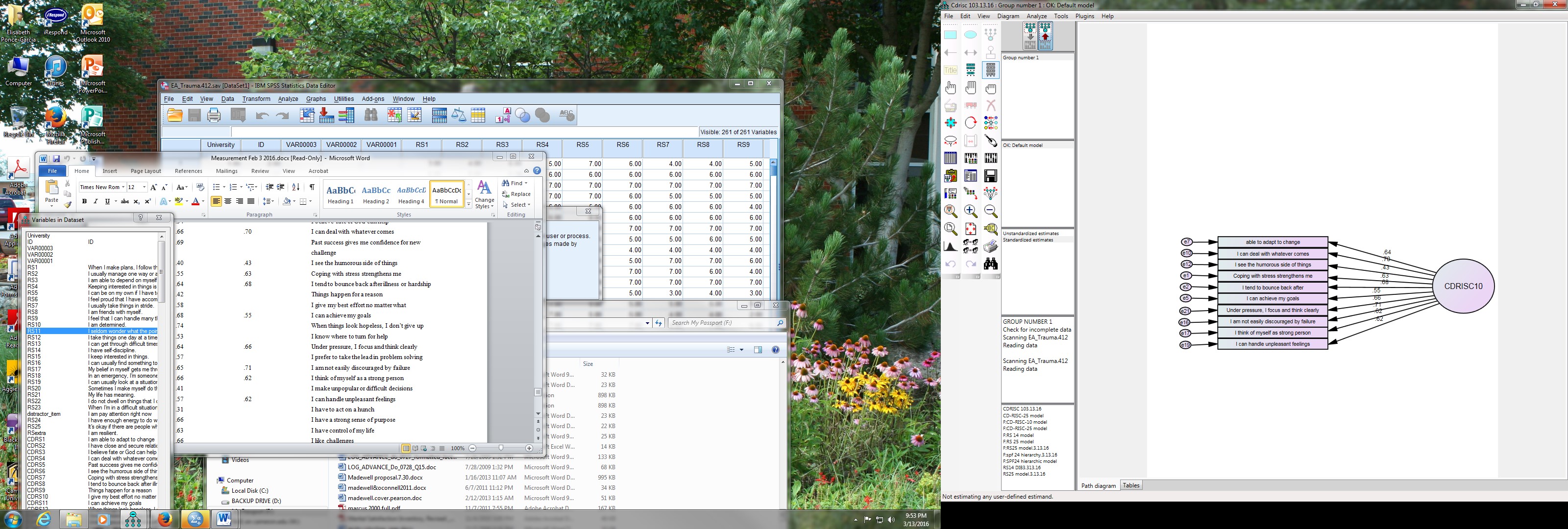 | | | |
| *Note.* Abbreviated items are presented in this table. To gain access to complete CD-RISC items, contact Dr. Jonathan Davidson at jonathan.davidson@duke.edu. | | | |

| Supplementary Figure 5. Hierarchic Factor Structure of the Scale of Protective Factors 24 | | | |
| --- | --- | --- | --- |
| **Error terms** | **Items** | **Covariates** | **Scale of Protective Factors (SPF) sub-scale names** |
| 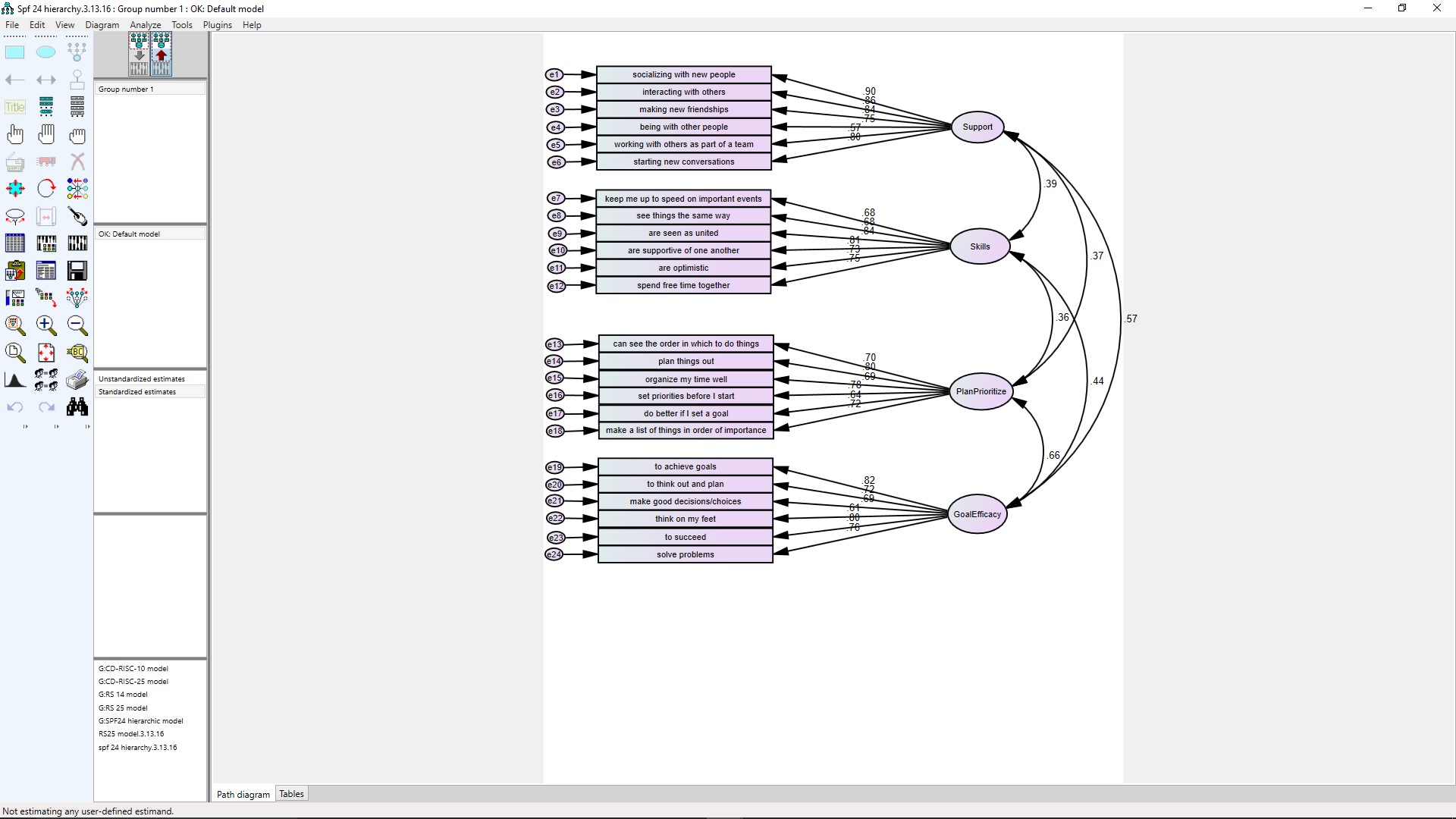 | | | |
| *Note.* Support denotes Social Support, Skills = Social Skills, PlanPrioritize = Planning and Prioritizing Behavior, and GoalEfficacy = Goal Efficacy. Together, these four protective factors denote the Scale of Protective Factors measure. Abbreviated items are presented in this table, for complete items refer to Ponce-Garcia, Madewell and Kennison (2016). | | | |
